# A Global Survey of Carbohydrate Esterase Families 1 and 10 in Oomycetes

**DOI:** 10.3389/fgene.2020.00756

**Published:** 2020-08-07

**Authors:** Sophie de Vries, Jan de Vries

**Affiliations:** ^1^Institute of Population Genetics, Heinrich Heine University Düsseldorf, Düsseldorf, Germany; ^2^Department of Applied Bioinformatics, Institute for Microbiology and Genetics, University of Göettingen, Göettingen, Germany; ^3^Göettingen Center for Molecular Biosciences (GZMB), University of Göettingen, Göettingen, Germany; ^4^Campus Institute Data Science, University of Göettingen, Göettingen, Germany

**Keywords:** oomycetes, CAZymes, evolution, gene families, plant pathogens, microbial lifestyle

## Abstract

Carbohydrate-active enzymes (CAZymes) are a cornerstone in the phytopathogenicity of filamentous microbes. CAZymes are required for every step of a successful infection cycle—from penetration, to nutrient acquisition (during colonization), to exit and dispersal. Yet, CAZymes are not a unique feature of filamentous pathogens. They are found across eukaryotic genomes and including, for example, saprotrophic relatives of major pathogens. Comparative genomics and functional analyses revealed that CAZyme content is shaped by a multitude of factors, including utilized substrate, lifestyle, and host preference. Yet, family size alone says little about usage. Indeed, in a previous study, we found that genes putatively coding for the CAZyme families of carbohydrate esterase (CE)1 and CE10, while not specifically enriched in number, were suggested to have lifestyle-specific gene expression patterns. Here, we used comparative genomics and a clustering approach to understand how the repertoire of the CE1- and CE10-encoding gene families is shaped across oomycete evolution. These data are combined with comparative transcriptomic analyses across homologous clusters within the gene families. We find that CE1 and CE10 have been reduced in number in biotrophic oomycetes independent of the phylogenetic relationship of the biotrophs to each other. The reduction in CE1 is different from that observed for CE10: While in CE10 specific clusters of homologous sequences show convergent reduction, CE1 reduction is caused by species-specific losses. Comparative transcriptomics revealed that some clusters of CE1 or CE10 sequences have a higher expression than others, independent of the species composition within them. Further, we find that CE1- and CE10-encoding genes are mainly induced in plant pathogens and that some homologous genes show lifestyle-specific gene expression levels during infection, with hemibiotrophs showing the highest expression levels.

## Introduction

Oomycetes include many destructive crop pathogens (Kamoun et al., 2015). Among the top 10 of these crop pathogens are many members of the genus *Phytophthora*. Next to plant pathogens, the diversity of oomycetes includes organisms that are pathogenic to animals (Phillips et al., 2008). Further, some oomycetes may not be pathogenic at all. A prime example for these are saprotrophic oomycetes (Marano et al., 2016; Thines, 2018). The oomycete pathogens can be broken down into biotrophs (that is, they require a living host to complete their life cycle), necrotrophs (that is, they kill their host to live from degradants), and hemibiotrophs (that is, they start as biotrophs and switch to necrotrophy at some later time point of infection) (Fawke et al., 2015).

Independent of their hosts and lifestyles, oomycetes need to degrade host tissue to colonize and/or make a living of the degradants. To do so, oomycetes use carbohydrate-active enzymes (CAZymes). Indeed, many CAZyme-encoding genes are induced during the infection process or colonization of dead tissue (Ah-Fong et al., 2017; Gaulin et al., 2018; de Vries et al., 2019; Grams et al., 2019). Further, many CAZymes are found in culture secrets (Wang et al., 2018), pointing to active secretion of these enzymes. Based on growth comparison on different carbohydrate sources, it was, however, hypothesized that some of the secreted CAZyme families of oomycete plant pathogens are more likely involved in plant pathogenesis rather than nutrient acquisition as respective carbon sources are poor substrates for *in vitro* growth (Brouwer et al., 2014).

CAZyme families are sorted into three major groups: glycoside hydrolases (GHs), polysaccharide lyases (PLs), and carbohydrate esterases (CEs). In addition, redox enzymes with auxiliary activities (AAs) and those with carbon-binding modules (CBMs) are required. Comparative genomics of filamentous pathogens strongly suggest that the different substrate compositions that the diversity of hosts offers shapes the requirement of different subfamilies of CAZymes (Ohm et al., 2012; Zhao et al., 2013; Gaulin et al., 2018; Shen et al., 2019). A study in fungi analyzing the role of substrate and lifestyle with regard to degradation capacity showed that first the substrate and second the lifestyle shaped the degradation profile (King et al., 2011). In a recent study by Barbi et al. (2020) that compared two saprotrophs, it was the transcriptomic response, especially that of CAZyme-encoding genes, that gave a good explanation for their different ecological strategies. Similarly, analyses of the fungus *Fusarium virguliforme* colonizing maize as an endophyte and soybean as a pathogen revealed that transcriptomic differences, also in CAZyme-encoding genes, are shaped by the mode of life of this fungus (Baetsen-Young et al., 2020). Comparisons of the transcriptome of a saprotrophic oomycete with those of plant pathogenic oomycetes also showed distinct expression of diverse CAZyme-encoding genes between the different oomycetes (de Vries et al., 2019). Among these CAZyme-encoding genes with lifestyle-specific expression were members of the family of CE1 and CE10.

Carbohydrate esterases are a large class of enzymes that remove ester-based modifications from carbohydrates (Cantarel et al., 2009). There are, depending on the classification, 16 recognized families of CEs; the assignment of the family CE10 to the group of CEs is currently challenged, as members of this family appear to act on ester-based modifications from other compounds than carbohydrates (Nakamura et al., 2017). With tens of thousands of CEs known from across the tree of life, the diversity of carbohydrate substrates is equally impressive—ranging from peptidoglycan that abounds in the bacterial cell wall, to chitin of fungi and insects, to pectin found in plants (Lombard et al., 2014; Nakamura et al., 2017).

In their entirety, the CEs are a versatile tool kit for a plant pathogen to get through different types of physical barriers that a plant body musters, including the cuticle, pectinaceous cell walls, and xylan (Ospino-Giraldo et al., 2010; van den Brink and de Vries, 2011; Zerillo et al., 2013; Nakamura et al., 2017). The substrate versatility of a given CE family varies, with the large families such as CE1 and CE4—as one would expect—having a larger number of described reactions on a given substrate (Nakamura et al., 2017).

Here we analyzed the CE1 and CE10 repertoire and their transcription in oomycetes with different host choices and lifestyles. In total, we screened 26 oomycetes. All oomycetes had genes of the families *CE1* and *CE10*. In addition, *in silico* prediction identified many species-specific composite enzymes for the two CAZyme families. The repertoire of CE1 and CE10 was significantly reduced in biotrophic pathogens; analyses of clusters of homologous sequences pinpoint two distinct mechanisms behind this reduction in biotrophs. We mapped gene expression levels, lifestyle information, and host choice onto each cluster within the phylogeny of CE1 and CE10 sequences. A few clusters of CE1 and CE10 were highly expressed during mycelial growth on plate or during infection. Additionally, we find some clusters of CE1 and CE10 that showed a higher expression in hemibiotrophs compared to biotrophs or necrotrophs. Our data pinpoint CE1 and CE10 homologs of oomycetes that likely have lifestyle-specific expression patterns.

## Materials and Methods

### Identification of Carbohydrate Esterase Families 1 and 10

We screened 26 oomycete datasets (25 genomes, one transcriptome; Supplementary Table S1; Tyler et al., 2006; Haas et al., 2009; Baxter et al., 2010; Lévesque et al., 2010; Kemen et al., 2011; Adhikari et al., 2013; Jiang et al., 2013; Quinn et al., 2013; Misner et al., 2015; Sambles et al., 2015; Sharma et al., 2015; Gaulin et al., 2018; Fletcher et al., 2019) for the presence of CE1 and CE10 family members. To do so, we initially used HMMER (biosequence analysis using profile hidden Markov models) on the dbCAN meta server (Yin et al., 2012). We then filtered the output data only using an e-value cutoff of 10^–5^. The filtered dataset was screened for sequences that are annotated as CE1, CE10, or either of the two in combination with another CAZyme family.

### Analyses of Gene Family Distribution

We tested whether the strict CE1 or CE10 families are enriched with regard to lifestyle (biotroph, hemibiotroph, necrotroph), host choice (plant or animal pathogen), and phylogenetic position (Peronosporales or Saprolegniales). Lifestyle, host choice, and phylogenetic position are summarized in Supplementary Table S2 according to Hughes and Grau (2007), Judelson (2012), Fawke et al. (2015), and Misner et al. (2015). For this analysis, we included only the genome data and excluded the data from the transcriptome of *Salisapilia sapeloensis.* The data were tested for normal distribution using a Shapiro–Wilk test (Shapiro and Wilk, 1965) and equal variance. All data were normally distributed. Depending on whether the compared datasets showed equal or unequal variance, a two-sample *t*-test or a Welch two-sample *t*-test was used to test for significant differences in CE1 and CE10 content. All statistical analyses were done in R v.3.6.0.

### Clustering of Homologous Groups of Carbohydrate Esterase 1 and 10 Sequences Utilizing a Phylogenetic Methodology

CE1 and CE10 sequences (that is, only protein sequences that contained either CE1 or CE10 domain and no other CAZyme domain) were aligned using MAFFT (Katoh and Standley, 2013). For CE1, we used G-INS-I, and for CE10, we used L-INS-I. We initially created full-length alignments for CE1 (Supplementary Dataset S1) and CE10 (Supplementary Dataset S2) protein sequences. Unsurprisingly, these had a very low relative identity in their amino acid sequences (length = 7,748 positions and 7.7% identity for CE1 and length = 5,026 positions and 10.4% identity for CE10). We therefore cropped the alignments (Supplementary Datasets S3, S4) to remove the highly variable N- and C-terminal regions and additionally removed CE10 sequences from the dataset that were too short. The curated datasets were realigned using G-INS-I for CE1 and L-INS-I for CE10. This resulted in an increase of ∼2% in identity rate (length = 1,523 positions and 9.9% identity for CE1 and length = 1,876 positions and 12.0% identity for CE10). Based on a trade-off between signal and relative amino acid identity, we used the full-length alignment for clustering CE1 sequences and the cropped alignment for clustering CE10 sequences into homologous sequence clusters. To do so, we used IQ-TREE (Nguyen et al., 2015) to create phylogenies using best model prediction (Kalyaanamoorthy et al., 2017). The phylogenies that we used for determining clusters are based on 100 bootstrap replicates. Bootstrap replicates were utilized as an additional guidance in pinpointing reasonable clusters of CE sequences.

### Calculating the Global and Local Identity of the Clusters of Carbohydrate Esterase 1 and 10 Sequences

Based on the clusters obtained utilizing the phylogenetic methodology outlined above, we extracted all full-length sequences of a given cluster, that is, 21 groups of CE1 sequences and 14 groups of CE10 sequences. We aligned these full-length sequences cluster by cluster using MAFFT (Katoh and Standley, 2013) with a G-INS-I approach, resulting in 21 alignments for CE1 and 14 alignments for CE10. We inspected these alignments using Geneious R11 and calculated average global pairwise identities. For several alignments, we additionally calculated average local pairwise identities.

### Expression Analyses

For expression analyses, we downloaded transcriptomic datasets from eleven oomycetes (*Albugo laibachii, Hyaloperonospora arabidopsidis, Plasmopara halstedii, Phytophthora parasitica, Phytophthora infestans, Pythium ultimum, Aphanomyces euteiches, Aphanomyces invadans, Aphanomyces astaci, Saprolegnia parasitica*, and *Saprolegnia diclina*; Supplementary Table S3; Jiang et al., 2013; Asai et al., 2014; Sharma et al., 2015; Ah-Fong et al., 2017; Prince et al., 2017; Gaulin et al., 2018). We used data from growing mycelium and late infection phases (or in case of the saprotroph *S. sapeloensis* late colonization phase). For *S. sapeloensis*, we used the TPM (Transcripts Per Million) values published in de Vries et al. (2019) (Supplementary Table S3). For the other datasets, we first assessed quality using FastQC v. 0.11.5^1^. We next removed the adapters and trimmed the raw read data using Trimmomatic v. 0.36 (Bolger et al., 2014). After reassessing the quality of the data, we used the trimmed read data and mapped them to the genomes of the respective oomycetes using Bowtie following calculation of TPM values via RSEM v. 1.2.18 (Li and Dewey, 2011).

Transcript budget (Supplementary Table S4) was calculated according to de Vries et al. (2018). In brief, the transcript budget is the relative amount of transcript invested into a given gene that is the percentage of the TPM normalized by the sum of all TPMs. To test for significant differences in expression levels between different sequence clusters, we used a Kruskal–Wallis test in combination with a *post hoc* Tukey test available in the R CRAN package PMCMR. For all pairwise comparisons of expression levels, normal distribution of the data was assessed using a Shapiro–Wilk test (Shapiro and Wilk, 1965). If data were not normally distributed, we calculated significant differences using a Mann–Whitney *U*-test (Mann and Whitney, 1947). Normally distributed data were additionally tested for equal variance. If the datasets had equal variance, a two-sample *t*-test was conducted. If the datasets showed unequal variance, a Welch two-sample *t*-test was used. We only compared differences in expression if at least three expression values were available per treatment.

To calculate fold changes (FCs), we used average TPM data from infection or colonization vs. mycelium and calculated log_2_ values.

## Results and Discussion

### Carbohydrate Esterases 1 and 10 Are Enriched in Hemibiotrophic and Necrotrophic Oomycetes

Filamentous pathogens require CEs to infect and colonize their hosts (Kubicek et al., 2014). Every host tissue has a unique fingerprint of carbohydrate and other ester compounds. The pathogens have to degrade these ester compounds in order to first overcome the physical barriers of host cells to facilitate infection and, second, to gain nutrients from them. Thus, depending on the lifestyle, a pathogen has different needs of its CE repertoire. Based on global differential gene expression patterns (de Vries et al., 2019), we pinpointed proteins that fall into the enzyme families CE1 and CE10 as lifestyle-specific candidates in oomycetes. To identify whether this is also reflected in the genomes of oomycete pathogens on a broader scale, we screened 25 genomes and one transcriptome of oomycetes with different host spectra and lifestyles.

**FIGURE 1:**
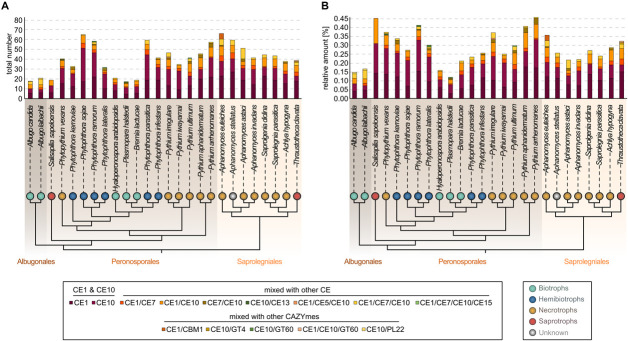
Distribution of carbohydrate esterase 1 (CE1)- and 10 (CE10)-coding genes among diverse oomycete genomes. On the top, a stacked column chart shows the coding capacity for the CE1 and CE10 families and composite enzymes with CE1 and CE10 domains found in 25 oomycete genomes and the transcriptome of *Salisapilia sapeloensis*; **(A)** the total number and **(B)** the relative coding capacity for CEs in relation to the total number of protein-coding genes on the genome—or in case of *S. sapeloensis* in relation to all the oomycete-specific protein-coding genes detected in the transcriptome. The stacked columns are projected onto a cladogram (drawn based on Diéguez-Uribeondo et al., 2009; Hulvey et al., 2010; McCarthy and Fitzpatrick, 2017; Fletcher et al., 2019) that shows the phylogenetic relationship between the oomycete species whose genomes/transcriptome were analyzed here. Note that alternatively, *S. sapeloensis* may also be placed between the genera *Phytophthora* and *Pythium* (Bennett and Thines, 2019). Background shading of the cladogram indicates the taxonomic orders (Albugonales, Peronosporales, and Saprolegniales). Colored bubbles indicate the lifestyles. Lifestyles were categorized according to Hughes and Grau (2007), Hulvey et al. (2010), Judelson (2012), Fawke et al. (2015), and Misner et al. (2015).

All 26 oomycetes encode CE1s and CE10s, as well as genes encoding different composite CE enzymes. Here, CE1 or CE10 domains are combined with either each other or CE5, CE7, CE13, or CE15 domains. The most abundant group of composition was CE1/CE10 (159 occurrences), followed by CE1/CE7 (70 occurrences) and CE1/CE7/CE10 (66 occurrences) (Figure 1A and Supplementary Table S5). Additionally, combinations with other CAZyme domains were identified (CBM1, GT4, GT60, and PL22). Both the CE combinations and the composite enzymes of CEs and other CAZyme domains appear to occur in a species-specific manner (Figure 1). Such species specificity likely is the result of the unique environment that each of the oomycetes dwells in.

Despite the occurrence of species-specific compositions of CEs, similar environmental settings may also shape the CE1 and CE10 repertoire. To analyze this, we, from now on, focus on genes encoding CE1 and CE10 members excluding combined CE1/CE10 and other composite enzymes. Indeed, we noted that genomes of biotrophic oomycetes and the transcriptome dataset of the saprotroph *S. sapeloensis* featured less CE1s and CE10s than the other oomycete genomes. Because *S. sapeloensis* is only represented by a transcriptome (and not a genome) and biotrophic oomycetes have less proteins encoded in their genomes compared to other oomycetes, we calculated the relative amount of CE1 and CE10 encoding sequences in respect to the total amount of sequences (Figure 1B). By doing so, we found that the skew for CE1- and CE10-encoding genes was still present for the biotrophic pathogens but not for the transcriptome of *S. sapeloensis.* This suggests that the lifestyle-dependent difference for biotrophs vs. other oomycete pathogens is robust, while that between *S. sapeloensis* and the other oomycetes may not be. Not inconsistent with the species-specific alterations in CE1 and CE10 repertoire, the appearance of this pattern supports that the overall CE repertoire might be shaped by the mode of life of a pathogenic oomycete.

**FIGURE 2:**
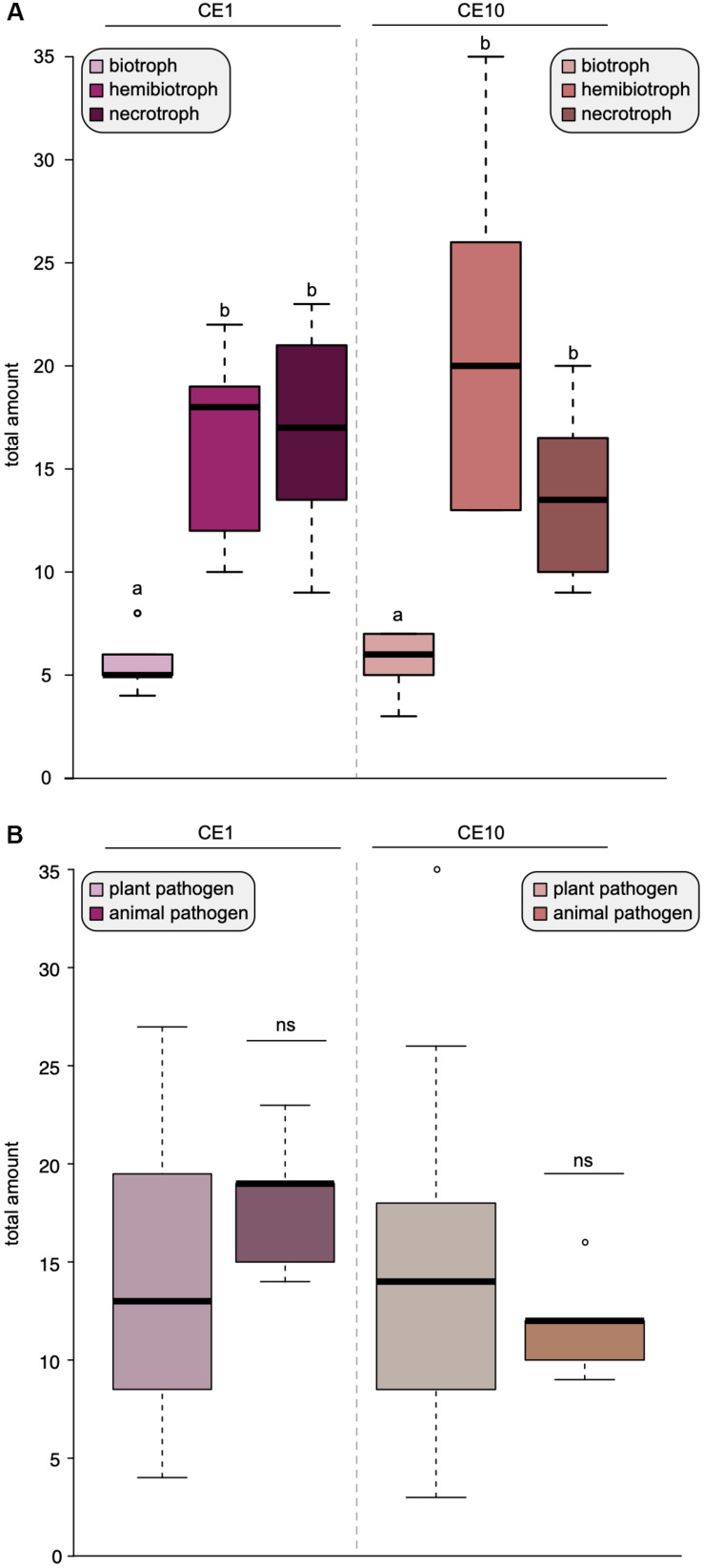
Lifestyles shape the distribution of carbohydrate esterases 1 (CE1) and 10 (CE10) in oomycetes. Box plots of the number of CE1- and CE10-coding genes found in 25 oomycete genomes. **(A)** The coding capacity for CE1 and CE10 sorted by lifestyles. **(B)** The coding capacity for CE1 and CE10 sorted by host type, i.e., whether the oomycete pathogens infect plants or animals. Significant differences (*p* < 0.05) are indicated by different letters, ns, not significant.

To investigate this further, we compared the difference between biotrophs, hemibiotrophs, and necrotrophs and found that biotrophs have a significantly reduced amount of both CE1- and CE10-encoding genes compared to necrotrophs and hemibiotrophs (Figure 2A). There was no significant difference in the overall repertoire of CE1 or CE10 between plant and animal pathogens (Figure 2B). This is noteworthy. Plants and animals have their own specific fingerprint of carbohydrate (and other) compounds. One would expect that the cocktail of compounds that an oomycete pathogen interacts with makes a bigger difference on its CE repertoire than the lifestyle. Indeed, comparisons of the genomes of animal and plant pathogenic *Aphanomyces* species indicated that the specialization to the different hosts drives a distinct CAZyme family repertoire (Gaulin et al., 2018). Also, in fungi, the substrate had a bigger influence on CAZyme content than lifestyle (Zhao et al., 2013). Yet, our data suggest that the opposite seems to be the case for oomycetes with regard to the CE families 1 and 10. Further, this pattern indicates that species-specific differences of CE1 and CE10 are likely found in the details—that is, specific homologous groups.

### Carbohydrate Esterase 1- and 10-Encoding Genes in Oomycetes Are Diverse and Bear Signs of a Dynamic Evolutionary History

**FIGURE 3:**
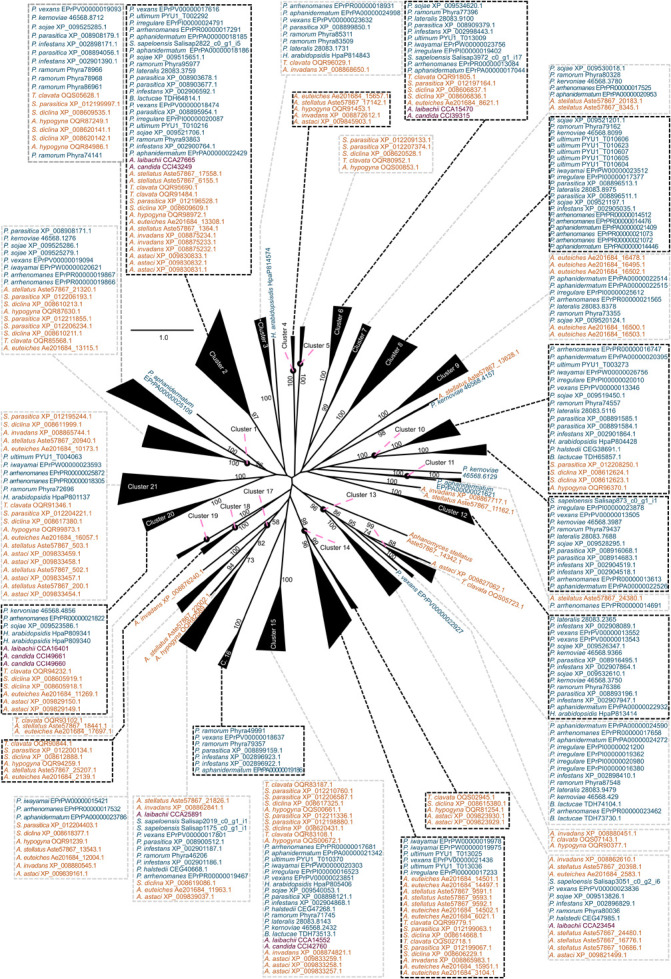
Clustering of the carbohydrate esterase 1 (CE1) gene family in oomycetes utilizing a phylogenetic approach. A maximum likelihood phylogeny of 383 CE1 protein sequences mined from 25 oomycete genomes and the transcriptome of *Salisapilia sapeloensis* is shown. The tree was computed based on a G-INS-I alignment using IQ-TREE multicore version 1.6.12; ModelFinder was used to test 168 protein models, and WAG+I+G4 was chosen according to the Bayesian information criterion (BIC) as the best model. One hundred bootstrap replicates were computed. Only bootstrap values ≥ 50 are shown. Colors indicate the phylogenetic affiliation of the species: blue, Peronosporales; orange, Saprolegniales; purple, Albugonales. Classification into orders is based on McCarthy and Fitzpatrick (2017). Alignment is provided in Supplementary Dataset S1.

**FIGURE 4:**
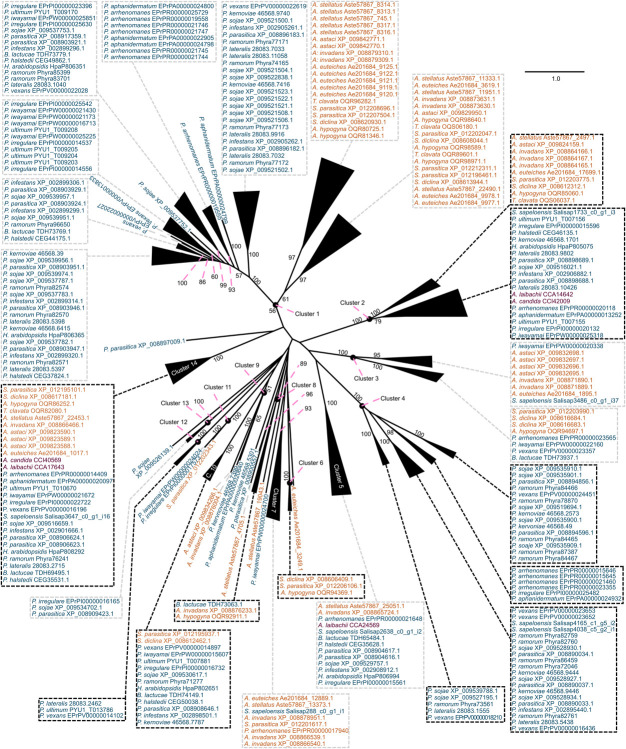
Clustering of the carbohydrate esterase 10 (CE10) gene family in oomycetes utilizing a phylogenetic approach. A maximum likelihood phylogeny of 313 CE10 protein sequences mined from 25 oomycete genomes and the transcriptome of *Salisapilia sapeloensis* is shown. The tree was computed based on an L-INS-I alignment using IQ-TREE multicore version 1.5.5; ModelFinder was used to test 144 protein models, and WAG+I+G4 was chosen according to the Bayesian information criterion (BIC) as the best model. One hundred bootstrap replicates were computed. Only bootstrap values ≥ 50 are shown. Colors indicate the phylogenetic affiliation of the species: blue, Peronosporales; orange, Saprolegniales; purple, Albugonales. Classification into orders is based on McCarthy and Fitzpatrick (2017). Alignment is provided in Supplementary Dataset S4.

What mechanisms shaped the repertoire of CE enzymes in oomycetes? We had a closer look at the CE1 and CE10 protein sequences of oomycetes. CE1 and CE10 sequences showed an overall low identity (CE1: 7.7% identity; cropped: 9.9% identity; CE10: 10.4% identity; cropped: 12.0% identity; Supplementary Datasets S1–S4). This is not surprising as these families are defined by nothing more than possessing at least one CE1 or CE10 domain, and the specific substrates of these enzymes differ. To explore the question on how the CE repertoire of oomycetes was shaped and to bring some reasonable order into their sequence diversity, we utilized a phylogenetic approach to separate the plethora of CE1 and CE10 sequences into clusters of homologous sequences (Figures 3, 4). This strategy allowed us to bring some structure into the largely uncharacterized range of CE1 and CE10 protein sequences; we used this structuring of the sequence diversity in CE1 and CE10 as a starting point for meaningful comparisons.

**FIGURE 5:**
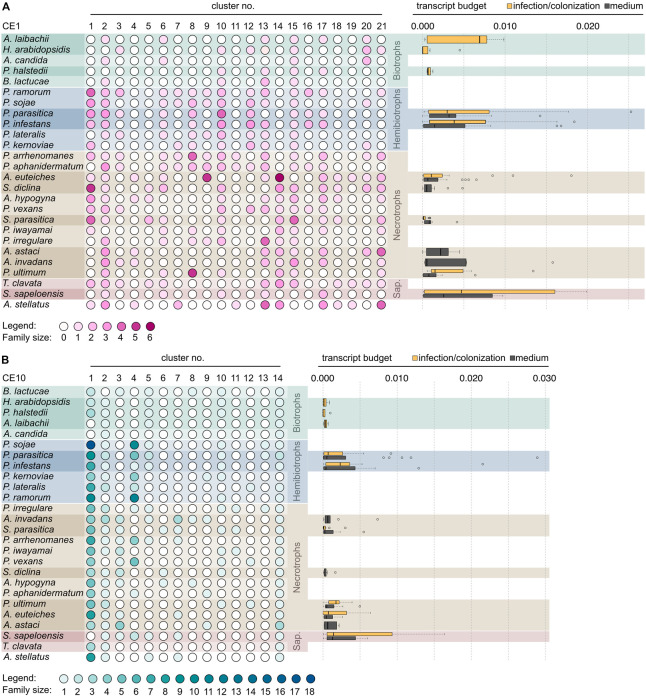
Distribution of clusters of the carbohydrate esterase 1 (CE1) and 10 (CE10) families across oomycetes. Heat map of gradient-colored dots that depicts the size of the clusters of the CE1 (**A**, pink) and CE10 (**B**, teal) gene families in 26 oomycetes. Cluster labels are based on Figures 3, 4. The darker a dot, the more genes were found in a given cluster. The species are clustered by lifestyle, followed by numbers of clusters present. The box plots to the right of the matrix give the expression as transcript budget of CE1- and CE10-encoding genes across each species. The yellow box plot shows the expression during infection or colonization (in case of the saprotroph *S. sapeloensis*), and gray box plots give expression during mycelial growth on artificial medium.

With our approach, we sorted the CE1 sequences into 21 clusters (Figure 3) and the CE10 sequences into 14 clusters (Figure 4). Within these groups, we found an average global identity of 43.0 ± 16.6% for CE1 and 40.3 ± 15.3% for CE10; and an average local identity of 47.6 ± 14.5% for CE1 and 45.3 ± 13.4% for CE10 (Supplementary Figures S1, S2). Most clusters in both families include sequences from species of Peronosporales and Saprolegniales, suggesting that these homologs have their origin in the last common ancestor of the later-branching oomycetes. However, not every species from the Peronosporales and Saprolegniales that were analyzed here is included in the aforementioned clusters (Figures 3, 4). Additionally, some clusters have more than one representative encoded in an oomycete genome. For example, homologs of CE10 cluster 2 are present in 24 of 25 oomycete genomes and the transcriptome of *S. sapeloensis* but is missing from *Bremia lactucae.* Given the phylogenetic position of *B. lactucae*, it appears that this oomycete has lost all CE10 sequences that we here assigned to cluster 2. Contrastingly, *A. invadans, P. ultimum*, and *Pythium irregulare* possess paralogs of CE10 sequences that fall into cluster 2 (Figure 4). Within the Albugonales, which only include biotrophic pathogens (Thines and Kamoun, 2010), we found that 15 of the 21 clusters of CE1 and 11 of 14 clusters of CE10 were missing (Figure 5). Given that the Albugonales are more closely related to the Peronosporales than to the Saprolegniales (McCarthy and Fitzpatrick, 2017), this suggests secondary loss of many of the CE1 and CE10 clusters in these biotrophs. In summary, the patterns within the clusters of homologous sequences support that the evolutionary history of CE1 and CE10 is a story of high dynamics. It is conceivable that multiple lineage-specific gains, convergent sequence evolution, and losses of several homologous groups gave rise to the rich sequence variation in CE1 and CE10 proteins that are encoded in the diverse oomycete genomes.

Secondary loss of entire clusters of CE1 and CE10 is apparent for all species. Yet, in case of the CE1 family, we find that the biotrophs in the dataset (*A. candida*, *A. laibachii*, *H. arabidopsidis*, *P. halstedii*, and *B. lactucae*) have retained the fewest numbers of clusters (Figure 5A)—speaking to a general reduction in CE1 sequence diversity. This might reflect the general streamlining of genomes of oomycete biotrophs (Spanu, 2012) that was previously already observed for other genes, such as those coding for proteases. A similar pattern, as observed in the biotrophs, was found for the saprotroph *S. sapeloensis*. As it stands, *S. sapeloensis* is, however, represented by just a transcriptome—and not a genome. It may be that sequences falling into some clusters were not expressed under the conditions in which *S. sapeloensis* was grown for obtaining the material for transcriptome sequencing. If the genome of the only other saprotroph in the dataset, *Thraustotheca clavata*, is any indication despite its distinct phylogenetic position, the genome of *S. sapeloensis* should indeed include more CE1 clusters. Future sequencing efforts for obtaining genomes of saprotrophic oomycetes will allow for circling back to the question of how frequent loss of genes coding for CE1s is in this lifestyle. The significantly reduced repertoire of CE1 in biotrophs, however, likely derives from the secondary loss of several clusters. The clusters that have been lost are different in the different species.

In the case of CE10, biotrophs have not lost more clusters than other species. Yet, they all have lost cluster 4, which is the cluster with the second highest number of sequences—especially in the hemibiotrophs (Figure 5B). Additionally, the biotrophs also have lost or reduced the number of CE10-encoding genes belonging to cluster 1 (Figure 5B). This might reflect not only the versatility in changes of the CE10 repertoire in the evolution of oomycetes but also the substrate versatility of CE10. Indeed, recently, it has been called into question whether CE10s should be classified as CEs as functional data suggest that several members act on non-carbohydrate substrates (Nakamura et al., 2017).

Overall, both genes encoding CE1 and CE10 proteins have been lost in biotrophic species. The underlying mechanisms by which this loss occurred, however, seem to be distinct from each other, with a species-specific reduction in CE1 and a convergent cluster-specific loss in CE10.

### Differential Gene Expression Patterns of Carbohydrate Esterase 1 and 10 Homologs Across 12 Oomycetes

Carbohydrate esterase-encoding genes are often among those genes that are induced during plant colonization by oomycetes (Ah-Fong et al., 2017; de Vries et al., 2019). Here, we asked how much transcript the different oomycetes invest into their *CE1* and *CE10* genes and whether specific clusters of the CE1 and CE10 families are specifically recruited during the infection process or mycelial growth on plate. For this, we analyzed transcriptome data of 12 oomycetes (including data from nine transcriptomic datasets from infection/tissue colonization and nine datasets from mycelial growth on plate).

**FIGURE 6:**
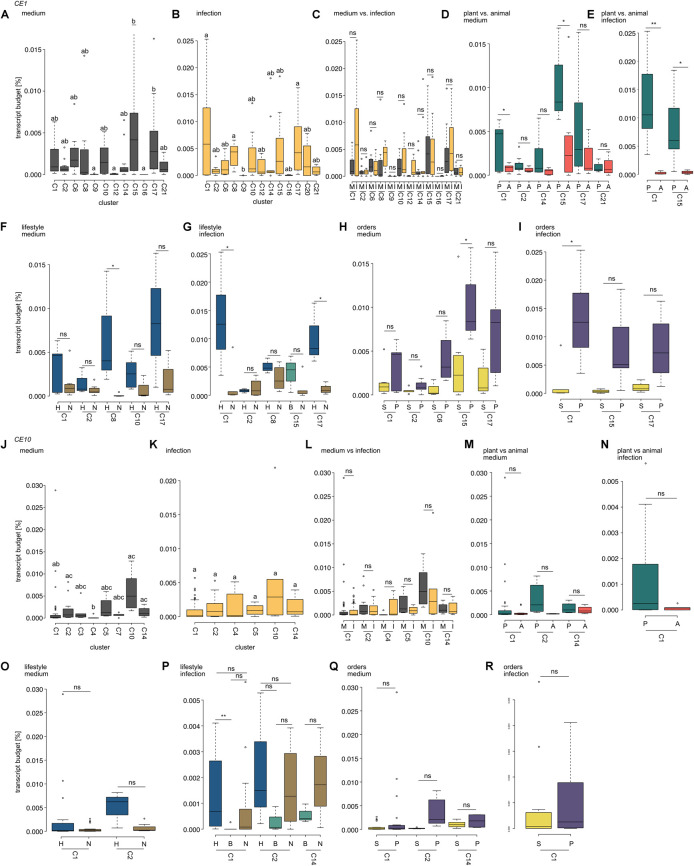
Comparison of expression levels of carbohydrate esterase 1 (CE1)- and 10 (CE10)-coding genes. Box plots show the transcript budget (relative to the overall expression level; calculations are based on TPM) that oomycetes invested into CE1- **(A–I)** and CE10-coding genes **(J–R)**. All cluster assignments are based on the phylogenies shown in Figure 3 (CE1) and Figure 4 (CE10). **(A,B,J,K)** Compare expression levels between the different clusters of the CE families analyzed for growth on plate (medium) and growth on a host (infection). Different letters indicate significant differences in expression levels (*p* < 0.05). **(C,I)** Show the comparison of expression levels between growth on medium (M) and during infection (I) within a cluster (ns, not significant). **(D,E,M,N)** Compare the expression levels between plant (P) and animal (A) pathogens during growth on medium **(D,M)** or infection **(E,N)**; significant differences are indicated by *p < 0.05 and **p < 0.01; ns, not significant. **(F,G,O,P)** Compare the expression levels between pathogens with different lifestyles (H, hemibiotroph; B, biotroph; and N, necrotroph) with each cluster for growth on medium or during infection. Significant differences are indicated by *p < 0.05, **p < 0.01, and ns, not significant. **(H,I,Q,R)** Show a comparison of expression levels between oomycetes from the orders of Saprolegniales **(S)** and Peronosporales **(P)**. Significant differences are indicated by *p < 0.05; non-significant differences are indicated by ns.

In agreement with the general reduction in CE10-encoding genes, we find that the biotrophs only invest minimal amounts of transcript in *CE10* across clusters, showing little variation (Figure 5B). In contrast, many of the hemibiotrophs and necrotrophs, as well as the saprotroph *S. sapeloensis*, show a broad spectrum of expression level of *CE10s*, with several genes having a rather high expression. Here, we analyzed the expression during the late infection phase in the hemibiotrophs and necrotrophs. The hemibiotrophs, necrotrophs, and the saprotrophs all were degrading plant material, while the biotrophs required a living host. The expression patterns we observed for CE10s may hence speak to an involvement of CE10s in plant degradation. The loss of CE10-encoding genes may thus be related to the low expression of these; that is, the biotrophs may lose what they do not use. This pattern is, however, not apparent in all samples. To investigate this in more detail, we next compared the expression of *CEs* in mycelium grown on plate vs. mycelium during an infection. To do so, we analyzed the transcript budget in percentage TPM across all testable clusters—that is, those clusters that entail at least three genes stemming from species for which transcriptome data were available. These criteria allowed for the comparison of 12 clusters (medium) and 13 clusters (infection) of the CE1 family and eight clusters (medium) and six clusters (infection) of the CE10 family (Figure 6).

Projecting gene expression data onto the individual clusters of homologs of CE1-encoding genes reveals that expression during mycelial growth on plate was significantly higher for clusters 15 and 17 compared to clusters 9, 12, and 16 (*p* < 0.05; Figure 6A). During infection, cluster 1 (*p* = 0.037), cluster 8 (*p* = 0.015), and cluster 17 (*p* = 0.017) had higher expression levels than cluster 9 (Figure 6B). Hence, only clusters 9 and 17 showed significant differences in expression in both mycelium and infection in CE1-encoding genes.

We used the same analysis for the gene repertoire of CE10-encoding genes (Figures 6J,K). Here, the expression pattern was similar for all testable clusters during infection. However, during growth on plate, we found that clusters 2 and 4 (*p* = 0.049) and clusters 4 and 14 (*p* = 0.017) significantly differed in their expression—clusters 2 and 14 exhibited an on average higher expression level than cluster 4. Additionally, cluster 10 had an on average significantly higher expression level than cluster 1 (*p* = 0.015) and cluster 4 (*p* = 0.001).

Our data suggest that, at least in case of CE1, different oomycetes recruit their own set of specific homologs of *CEs* during infection. Given that the different clusters have not the exact same species setup due to lineage-specific expansions and losses, we next asked whether these cluster-specific expression patterns may correlate with lifestyle specificity, host choice, and/or the species composition within those clusters. Therefore, we next investigated the cluster composition of the clusters that differed significantly in their relative expression levels.

CE1 clusters 9 and 12 only consist of sequences from plant pathogenic oomycetes (cluster 9 includes only *A. euteiches*, cluster 12 consists of several plant pathogenic oomycetes from the Peronosporales with different lifestyles), while clusters 15 and 17 include sequences from a mixture of plant and animal pathogenic (and saprotrophic in cluster 17) oomycetes from the three orders of later-branching oomycetes (Figure 3). Hence, clusters 9 and 12 differ in two ways from clusters 15 and 17: They are less diverse in (i) their species compositions and (ii) the host/substrate of the included species. In CE10, clusters 2 and 14 include sequences from Peronosporales, Albugonales, and Saprolegniales; clusters 1 and 10 include Peronosporales and Saprolegniales; and cluster 4 includes only Peronosporales (Figure 4). Further, clusters 2 and 14 include the saprotroph *S. sapeloensis*, as well as plant and animal pathogenic oomycetes. Similarly, clusters 1 and 10 also include representatives of animal and plant pathogens. Cluster 4, however, lacks genes from animal pathogenic Saprolegniales. Despite these differences, neither in the clusters from CE1 nor CE10, it is the species composition that can explain the differences in expression levels (Supplementary Table S4). It is rather that all species in clusters 15 and 17 (CE1) and clusters 2 and 14 and cluster 10 (CE10) (even those that overlap between the significantly different clusters) have a consistently higher expression level than clusters 9 and 12 (CE1) and cluster 4 and cluster 1 (CE10), respectively. That said, we observe some variance in the expression between genes from different species within all testable clusters.

### Plant Pathogens Induce Carbohydrate Esterase 1- and 10-Encoding Genes During Infection

Next, we asked whether some *CE1* or *CE10* clusters show a general higher expression during infection or mycelial growth across a specific cluster. This would suggest that a specific set of *CE1s* or *CE10s* has a tendency to be recruited during infection or mycelial growth on plate by several distinct pathogens. To tackle this question, we analyzed clusters that entailed at least three genes for which we had transcriptomic data available. We found that none of the clusters that could be analyzed (12 in CE1 and six in CE10) showed a significant difference between infection and mycelium (Figures 6C,L). This suggests that most CE1 and CE10 homologs are used either in a species-specific manner during infection or only for pathogens with certain similar characteristics (e.g., plant vs. animal pathogen, lifestyle, or phylogenetic relatedness). To investigate this more closely, we compared FCs during growth on plate and infection/substrate colonization for four plant pathogens (*P. infestans*, *P. parasitica*, *P. ultimum*, and *A. euteiches*), one animal pathogen (*S. parasitica*), and one saprotroph (*S. sapeloensis*). Genes were defined as induced during infection/colonization when they had a log_2_(FC) ≥ 1 for infection/colonization vs. growth on plate and as reduced when they had a log_2_(FC) ≤ −1 (Table 1).

**TABLE 1:** Differential gene expression changes for carbohydrate esterase 1 (CE1)- and 10 (CE10)-encoding genes calculated as log_2_[fold change (FC)] for growth during infection/colonization vs. growth on medium.

**CE1**					**CE10**				
**Species**	**Cluster**	**Gene ID**	**Protein ID**	**Log2(FC)**	**Species**	**Cluster**	**Gene ID**	**Protein ID**	**Log2(FC)**
*A. euteiches*	1	Ae201684_13115.1	Ae201684_13115.1	0.71	*A. euteiches*	1	Ae201684_3619.1	Ae201684_3619.1	**1.37**
*P. infestans*	1	PITG_15956T0	XP_002898171.1	−0.84	*A. euteiches*	1	Ae201684_9119.1	Ae201684_9119.1	NA
*P. infestans*	1	PITG_11920T0	XP_002901390.1	**4.84**	*A. euteiches*	1	Ae201684_9120.1	Ae201684_9120.1	NA
*P. parasitica*	1	PPTG_03128	XP_008894056.1	**1.80**	*A. euteiches*	1	Ae201684_9121.1	Ae201684_9121.1	0.26
*P. parasitica*	1	PPTG_12733	XP_008908171.1	0.76	*A. euteiches*	1	Ae201684_9122.1	Ae201684_9122.1	NA
*P. parasitica*	1	PPTG_12738	XP_008908179.1	**6.95**	*A. euteiches*	1	Ae201684_9125.1	Ae201684_9125.1	**1.64**
*S. parasitica*	1	KDO29501	XP_012199997.1	−**3.06**	*A. euteiches*	1	Ae201684_9977.1	Ae201684_9977.1	0.46
*S. parasitica*	1	KDO23081	XP_012206193.1	**2.12**	*A. euteiches*	1	Ae201684_9978.1	Ae201684_9978.1	NA
*S. parasitica*	1	KDO23124	XP_012206234.1	−**3.83**	*P. infestans*	1	PITG_07333T0	XP_002905261.1	0.50
*S. parasitica*	1	KDO17433	XP_012211855.1	−**3.04**	*P. infestans*	1	PITG_07334T0	XP_002905262.1	0.99
*A. euteiches*	2	Ae201684_13308.1	Ae201684_13308.1	0.43	*P. infestans*	1	PITG_14190T0	XP_002899296.1	**1.54**
*P. infestans*	2	PITG_12144T0	XP_002900764.1	0.68	*P. infestans*	1	PITG_14194T0	XP_002899299.1	**8.00**
*P. infestans*	2	PITG_03525T0	XP_002906592.1	0.17	*P. infestans*	1	PITG_14206T0	XP_002899306.1	0.67
*P. parasitica*	2	PPTG_03921	XP_008895954.1	−**1.48**	*P. infestans*	1	PITG_14215T0	XP_002899314.1	−**1.35**
*P. parasitica*	2	PPTG_10149	XP_008903677.1	−**1.14**	*P. infestans*	1	PITG_14222T0	XP_002899320.1	0.85
*P. parasitica*	2	PPTG_10149	XP_008903678.1	−**1.14**	*P. parasitica*	1	PPTG_04107	XP_008896182.1	**1.49**
*P. ultimum*	2	PYU1_T002292	PYU1_T002292	0.91	*P. parasitica*	1	PPTG_04108	XP_008896183.1	−**1.44**
*P. ultimum*	2	PYU1_T010216	PYU1_T010216	0.97	*P. parasitica*	1	PPTG_09884	XP_008903921.1	−**2.77**
*S. parasitica*	2	KDO32877	XP_012196528.1	NA	*P. parasitica*	1	PPTG_09887	XP_008903924.1	−**4.15**
*S. sapeloensis*	2	Salisap2822_c0_g1_i5	Salisap2822_c0_g1_i5	**1.13**	*P. parasitica*	1	PPTG_09892	XP_008903929.1	NA
*P. parasitica*	3	PPTG_07423	XP_008899850.1	−0.77	*P. parasitica*	1	PPTG_09908	XP_008903946.1	−**3.47**
*A. euteiches*	4	Ae201684_15657.1	Ae201684_15657.1	0.59	*P. parasitica*	1	PPTG_09909	XP_008903947.1	**1.77**
*S. parasitica*	5	KDO21933	XP_012207374.1	−**4.24**	*P. parasitica*	1	PPTG_09913	XP_008903951.1	−**3.06**
*S. parasitica*	5	KDO20180	XP_012209133.1	−**2.75**	*P. parasitica*	1	PPTG_20345	XP_008917359.1	NA
*A. euteiches*	6	Ae201684_8621.1	Ae201684_8621.1	0.69	*P. ultimum*	1	PYU1_T009170	PYU1_T009170	0.66
*P. infestans*	6	PITG_06289T0	XP_002998443.1	**1.07**	*P. ultimum*	1	PYU1_T009203	PYU1_T009203	−0.87
*P. parasitica*	6	PPTG_14144	XP_008909379.1	−**2.02**	*P. ultimum*	1	PYU1_T009204	PYU1_T009204	−0.50
*P. ultimum*	6	PYU1_T013009	PYU1_T013009	−0.56	*P. ultimum*	1	PYU1_T009205	PYU1_T009205	**1.56**
*S. parasitica*	6	KDO31968	XP_012197164.1	**1.55**	*P. ultimum*	1	PYU1_T009208	PYU1_T009208	**2.48**
*S. sapeloensis*	6	Salisap3972_c0_g1_i17	Salisap3972_c0_g1_i17	0.31	*S. parasitica*	1	KDO16981	XP_012212311.1	NA
*P. infestans*	8	PITG_07069T0	XP_002905035.1	**1.32**	*S. parasitica*	1	KDO20570	XP_012208696.1	NA
*P. parasitica*	8	PPTG_04363	XP_008896511.1	−**1.16**	*S. parasitica*	1	KDO21831	XP_012207504.1	NA
*P. parasitica*	8	PPTG_04365	XP_008896513.1	−0.18	*S. parasitica*	1	KDO27271	XP_012202047.1	−**1.87**
*P. ultimum*	8	PYU1_T010604	PYU1_T010604	**7.58**	*S. parasitica*	1	KDO32805	XP_012196461.1	−**2.02**
*P. ultimum*	8	PYU1_T010605	PYU1_T010605	**4.19**	*A. euteiches*	2	Ae201684_17699.1	Ae201684_17699.1	0.45
*P. ultimum*	8	PYU1_T010606	PYU1_T010606	NA	*P. infestans*	2	PITG_03840T0	XP_002906882.1	−0.24
*P. ultimum*	8	PYU1_T010607	PYU1_T010607	0.49	*P. parasitica*	2	PPTG_06514	XP_008898688.1	−**2.43**
*P. ultimum*	8	PYU1_T010623	PYU1_T010623	**5.46**	*P. parasitica*	2	PPTG_06515	XP_008898689.1	−**1.80**
*A. euteiches*	9	Ae201684_16478.1	Ae201684_16478.1	NA	*P. ultimum*	2	PYU1_T007155	PYU1_T007155	0.55
*A. euteiches*	9	Ae201684_16495.1	Ae201684_16495.1	**1.08**	*P. ultimum*	2	PYU1_T007156	PYU1_T007156	0.40
*A. euteiches*	9	Ae201684_16500.1	Ae201684_16500.1	NA	*S. parasitica*	2	KDO25553	XP_012203775.1	NA
*A. euteiches*	9	Ae201684_16502.1	Ae201684_16502.1	NA	*S. sapeloensis*	2	Salisap1733_c0_g1_i3	Salisap1733_c0_g1_i3	−0.50
*A. euteiches*	9	Ae201684_16503.1	Ae201684_16503.1	NA	*A. euteiches*	3	Ae201684_1895.1	Ae201684_1895.1	0.57
*P. infestans*	10	PITG_11087T0	XP_002901864.1	−0.53	*S. parasitica*	3	KDO25340	XP_012203990.1	**3.63**
*P. infestans*	10	PITG_07530T0	XP_002904518.1	0.01	*S. sapeloensis*	3	Salisap3486_c0_g1_i37	Salisap3486_c0_g1_i37	0.49
*P. infestans*	10	PITG_07531T0	XP_002904519.1	0.07	*P. infestans*	4	PITG_20786T0	XP_002895440.1	**2.73**
*P. parasitica*	10	PPTG_02279	XP_008891585.1	−**1.18**	*P. parasitica*	4	PPTG_00395	XP_008890033.1	NA
*P. parasitica*	10	PPTG_18390	XP_008914683.1	−0.53	*P. parasitica*	4	PPTG_00396	XP_008890034.1	**3.50**
*P. parasitica*	10	PPTG_24575	XP_008916068.1	**1.11**	*P. parasitica*	4	PPTG_00399	XP_008890037.1	**5.64**
*P. ultimum*	10	PYU1_T003273	PYU1_T003273	**2.50**	*P. parasitica*	4	PPTG_03658	XP_008894596.1	NA
*S. parasitica*	10	KDO21071	XP_012208250.1	−**1.08**	*P. parasitica*	4	PPTG_21355	XP_008894856.1	NA
*S. sapeloensis*	10	Salisap873_c0_g1_i1	Salisap873_c0_g1_i1	0.68	*S. sapeloensis*	4	Salisap4038_c5_g2_i1	Salisap4038_c5_g2_i1	−0.94
*P. infestans*	12	PITG_01186T0	XP_002907864.1	**5.43**	*S. sapeloensis*	4	Salisap4165_c1_g5_i2	Salisap4165_c1_g5_i2	−0.62
*P. infestans*	12	PITG_01274T0	XP_002907947.1	NA	*P. infestans*	5	PITG_00294T0	XP_002908912.1	0.17
*P. infestans*	12	PITG_01423T0	XP_002908089.1	**3.48**	*P. parasitica*	5	PPTG_10879	XP_008904617.1	0.37
*P. parasitica*	12	PPTG_01585	XP_008893196.1	NA	*S. sapeloensis*	5	Salisap2638_c0_g1_i2	Salisap2638_c0_g1_i2	0.83
*P. parasitica*	12	PPTG_19740	XP_008916495.1	**4.32**	*S. parasitica*	6	KDO23154	XP_012206106.1	**2.59**
*P. infestans*	13	PITG_15466T0	XP_002898410.1	**1.15**	*A. euteiches*	7	Ae201684_12889.1	Ae201684_12889.1	**2.31**
*A. euteiches*	14	Ae201684_14497.1	Ae201684_14497.1	0.61	*S. parasitica*	7	KDO27748	XP_012201617.1	−0.59
*A. euteiches*	14	Ae201684_14501.1	Ae201684_14501.1	**1.04**	*S. sapeloensis*	7	Salisap288_c0_g1_i1	Salisap288_c0_g1_i1	**2.45**
*A. euteiches*	14	Ae201684_14502.1	Ae201684_14502.1	0.60	*P. infestans*	10	PITG_14598T0	XP_002898501.1	0.74
*A. euteiches*	14	Ae201684_15951.1	Ae201684_15951.1	−0.03	*P. parasitica*	10	PPTG_13924	XP_008908646.1	−0.73
*A. euteiches*	14	Ae201684_3104.1	Ae201684_3104.1	**1.19**	*P. ultimum*	10	PYU1_T007881	PYU1_T007881	−0.79
*A. euteiches*	14	Ae201684_6021.1	Ae201684_6021.1	**1.47**	*S. parasitica*	10	KDO33174	XP_012195937.1	−**4.44**
*P. ultimum*	14	PYU1_T013025	PYU1_T013025	−0.92	*S. parasitica*	11	KDO26962	XP_012202343.1	−**2.70**
*P. ultimum*	14	PYU1_T013036	PYU1_T013036	−0.45	*P. ultimum*	12	PYU1_T013786	PYU1_T013786	**1.28**
*S. parasitica*	14	KDO30261	XP_012199063.1	NA	*P. parasitica*	13	PPTG_14185	XP_008909423.1	−0.02
*S. parasitica*	14	KDO30265	XP_012199067.1	0.04	*A. euteiches*	14	Ae201684_1017.1	Ae201684_1017.1	**1.40**
*P. infestans*	15	PITG_06891T0	XP_002904868.1	0.13	*P. infestans*	14	PITG_10850T0	XP_002901666.1	**3.00**
*P. parasitica*	15	PPTG_06099	XP_008898121.1	0.44	*P. parasitica*	14	PPTG_12375	XP_008906624.1	−**2.74**
*P. ultimum*	15	PYU1_T010370	PYU1_T010370	−0.33	*P. ultimum*	14	PYU1_T010670	PYU1_T010670	**1.66**
*S. parasitica*	15	KDO30514	XP_012198880.1	NA	*S. parasitica*	14	KDO34365	XP_012195101.1	−**5.21**
*S. parasitica*	15	KDO22670	XP_012206587.1	NA	*S. sapeloensis*	14	Salisap3647_c0_g1_i16	Salisap3647_c0_g1_i16	0.44
*S. parasitica*	15	KDO18535	XP_012210760.1	−0.95					
*S. parasitica*	15	KDO17959	XP_012211336.1	−**3.67**					
*P. infestans*	16	PITG_16693T0	XP_002896922.1	NA					
*P. infestans*	16	PITG_16694T0	XP_002896923.1	NA					
*P. parasitica*	16	PPTG_06868	XP_008899159.1	NA					
*A. euteiches*	17	Ae201684_11963.1	Ae201684_11963.1	−0.30					
*A. euteiches*	17	Ae201684_12004.1	Ae201684_12004.1	0.39					
*P. infestans*	17	PITG_22946T0	XP_002901186.1	−0.33					
*P. infestans*	17	PITG_22947T0	XP_002901187.1	**1.16**					
*P. parasitica*	17	PPTG_07887	XP_008900512.1	**2.55**					
*S. parasitica*	17	KDO24943	XP_012204403.1	0.06					
*S. sapeloensis*	17	Salisap1175_c0_g1_i1	Salisap1175_c0_g1_i1	0.42					
*S. sapeloensis*	17	Salisap2019_c0_g1_i1	Salisap2019_c0_g1_i1	0.27					
*A. euteiches*	18	Ae201684_2139.1	Ae201684_2139.1	0.79					
*S. parasitica*	18	KDO29241	XP_012200134.1	−**5.58**					
*A. euteiches*	19	Ae201684_17697.1	Ae201684_17697.1	**1.27**					
*A. euteiches*	20	Ae201684_11269.1	Ae201684_11269.1	−0.80					
*A. euteiches*	21	Ae201684_10173_1	Ae201684_10173.1	0.16					
*A. euteiches*	21	Ae201684_16057_1	Ae201684_16057.1	**1.38**					
*P. ultimum*	21	PYU1_T004063	PYU1_T004063	**1.44**					
*S. parasitica*	21	KDO34207	XP_012195244.1	−**1.89**					
*S. parasitica*	21	KDO25153	XP_012204221.1	−**1.89**					

*Values are written in bold font if the Log_2_(fold change) is larger than 1 or smaller than −1.*

For the *CE1* family, we were able to calculate log_2_(FC) for genes from 18 clusters. In all 18 clusters, genes with no induction or reduction were found (Table 1). Of those clusters where genes with log_2_(FC) ≥ 1 or log_2_(FC) ≤ −1 were identified, six clusters had both genes with induced and reduced gene expression, six other clusters had only genes that were induced, and three clusters had only genes with reduced gene expression. Most genes showed a log_2_(FC) ≥ 1 (26 induced, 15 reduced), suggesting that most of the CE1-encoding genes, which are differentially responding, are induced during late infection. This was especially true for the plant pathogens, where only *P. parasitica* has CE1-encoding genes that are reduced during the infection process, while the other plant pathogens (even *A. euteiches*, which is a saprolegnian oomycete), only have CE1-encoding genes, which were induced during plant infection. The animal pathogen *S. parasitica* has both induced and reduced CE1-encoding genes, although those with a reduction in gene expression are more abundant (two induced, 10 reduced). It should, however, be considered that *S. parasitica* is the only animal pathogen with a genome for which data for growth on mycelium and growth during infection were available at the time of the analyses. Therefore, we cannot assume that the pattern observed in *S. parasitica* represents that of other animal pathogenic oomycetes. Future transcriptomic data on other animal pathogenic oomycetes will highlight whether this pattern is valid across the animal pathogens.

For the *CE10* family, we could calculate log_2_(FC) for genes from 12 clusters. All 12 clusters include genes that showed neither an induction nor a reduction in gene expression during infection. Of those clusters that include genes showing a log_2_(FC) ≥ 1 or log_2_(FC) ≤ −1 in the comparison of growth during infection vs. growth on plate, two clusters included genes that were either induced or reduced, five clusters included only induced genes, and one cluster included only downregulated genes. Most of the induced genes (16 of 19 differentially responding genes) are from plant pathogens, one is from the saprotroph *S. sapeloensis* and two are from *S. parasitica*. In contrast, only the two plant pathogens *P. parasitica* (eight genes) and *P. infestans* (one gene) and the animal pathogen *S. saprolegnia* (five genes) had genes with reduced gene expression.

Both *CE1* and *CE10* appear to be more often induced during infection of plants than during the infection of the animal host analyzed in this study. While we cannot infer a general pattern of expression of *CE1* and *CE10* genes (due to the lack of available data), our data suggest that most plant pathogens preferentially induce their CE1- and CE10-encoding genes during infection.

### Clusters of Carbohydrate Esterases 1 and 10 Show Lifestyle-Specific Expression Levels During Infection

In our aforementioned analyses, we noted that some of the clusters for which we compared the transcript budget showed variances of their gene expression levels depending on whether they stem from a plant or animal pathogen. Indeed, CAZymes, including CEs, are employed during tissue colonization and nutrient acquisition by pathogens and saprotrophs (Kubicek et al., 2014). Comparative genomics and transcriptomics have highlighted a differential use of these enzymes depending on the hosts and lifestyles of filamentous microorganisms (Ohm et al., 2012; Gaulin et al., 2018; de Vries et al., 2019; Shen et al., 2019; Baetsen-Young et al., 2020; Barbi et al., 2020).

To test whether host choice or lifestyle may have an impact on *CE* expression levels, we compared the transcript budget of CE-encoding genes from oomycetes with different host choices (plant or animal pathogen) and different lifestyles (biotrophs, hemibiotrophs, or necrotrophs) during growth on plate or infection (Figures 6D–G,M–P). As a control, we analyzed the role of the evolutionary relationship of oomycetes (Peronosporales vs. Saprolegniales; Figures 6H,I,Q,R) on differences in transcript budget during growth on plate or during an infection.

We found that genes in CE1 clusters 1 and 17 show significantly higher expression during infection in the hemibiotrophic pathogens compared to the necrotrophic ones in this cluster (Figures 6F,G). Similarly, genes from CE1 cluster 8 show significantly higher expression in the mycelium of hemibiotrophs vs. necrotrophs. For CE10, hemibiotrophs invest a significantly higher transcript budget in genes from cluster 1 than biotrophs during infection (Figure 6P). This is in agreement with the previously observed differences in some putative orthologs of *CE1* and *CE10* genes between the saprotroph *S. sapeloensis* and diverse plant pathogenic oomycetes (de Vries et al., 2019).

Genes that fall in CE1 clusters 1 and 15 show a higher expression during growth on medium or host in plant pathogens compared to animal pathogens (Figures 6D,E). This trend is not apparent in any testable cluster from the CE10 family (Figures 6M,N). The difference in expression during infection for genes in CE1 cluster 1 and the differences in mycelial expression while growing on medium for genes in CE1 cluster 15 are, however, also visible when Peronosporales are compared to Saprolegniales, leaving a possibility that this change in expression is phylogenetically related (Figures 6H,I). More transcriptomic data on animal pathogens from the Peronosporales and plant pathogens from the Saprolegniales will be useful to determine whether the expression of *CEs* (and many other genes) is rather determined by relatedness or environment. Given that transcriptomic datasets of plant pathogens (oomycetes and fungi) paint a picture of environmentally dependent and highly versatile transcription within one organism (e.g., Ah-Fong et al., 2017, 2019; Gaulin et al., 2018; Baetsen-Young et al., 2020), it is likely that convergent expression patterns in different species with similar environments exist. First data that this happens already exist (Barbi et al., 2020).

## Data Availability Statement

All datasets presented in this study are included in the article/Supplementary Material. Further, all genome and transcriptome data used is publically available; the accession numbers can be found in the Supplementary Material.

## Author Contributions

SV conceived of the study. SV and JV carried out the analyses, discussed the content, and wrote the manuscript. All authors contributed to the article and approved the submitted version.

## Conflict of Interest

The authors declare that the research was conducted in the absence of any commercial or financial relationships that could be construed as a potential conflict of interest.

## Footnotes

1 www.bioinformatics.babraham.ac.uk/projects/fastqc
