# Supplementary material for: A Global Survey of Carbohydrate Esterase Families 1 and 10 in Oomycetes
Source: Front Genet. 2020 Aug 7;11:756. doi: 10.3389/fgene.2020.00756 (PMC7427535; doi:10.3389/fgene.2020.00756)
Supplement: Supplementary file 1 [file Table_1.docx]

**Table S1. Predicted protein and transcript datasets of oomycete genomes and the *S. sapeloensis* transcriptome assembly used in this study.**

| **Organism** | **Type of data** | **Reference** | **Accession/**  **Availability** |
| --- | --- | --- | --- |
| *Saprolegnia*  *parasitica* CBS 223.65 | genome (cDNA & protein sequences) | NCBI GenBank;  Jiang et al. 2013; PLoS Genet 9: e1003272 | PRJNA280969PRJNA36583 |
| *Saprolegnia diclina*VS20 | genome (protein sequences) | NCBI GenBank | PRJNA255245PRJNA86859 |
| *Aphanomyces*  *invadans* NJM9701 | genome (protein sequences) | NCBI GenBank | PRJNA258292PRJNA188082 |
| *Aphanomyces astaci* APO3 | genome (protein sequences) | NCBI GenBank | PRJNA264335 PRJNA187372 |
| *Aphanomyces euteiches* ATCC201684 | genome (CDS & protein sequences) | AphanoDB;  Gaulin et al. 2018 | Aphanomyces_euteichesV3 |
| *Aphanomyces stellatus*  Strain 57867 | genome (protein sequences) | AphanoDB;  Gaulin et al. 2018 | Aste57867 |
| *Achlya hypogyna*ATTC 48635 | genome (protein sequences) | NCBI GenBank; Misner et al. 2015; Genome Biol Evol 7:120-135 | PRJNA169234 |
| *Thraustotheca*  *clavata*ATCC 34112 | genome (protein sequences) | NCBI GenBank; Misner et al. 2015; Genome Biol Evol 7:120-135 | PRJNA169235 |
| *Pythium irregulare* DAOM BR486 | genome (protein sequences) | Ensembl Protists (Downloads); Adhikari et al. 2013; PLoS One 8: e75072 | pir_scaffolds_v1 |
| *Pythium iwayamai* DAOM BR242034 | genome (protein sequences) | Ensembl Protists (Downloads); Adhikari et al. 2013; PLoS One 8: e75072 | piw_scaffolds_v1 |
| *Pythium ultimum* DAOM BR144 | genome (protein sequences) | Ensembl Protists (Downloads); Lévesque et al. 2010; Genome Biology 11:R73 | pug |
| *Pythium*  *aphanidermatum* DAOM BR444 | genome (protein sequences) | Ensembl Protists (Downloads); Adhikari et al. 2013; PLoS One 8: e75072 | pag1_scaffolds_v1 |
| *Pythium*  *arrhenomanes* ATCC 12531 | genome (protein sequences) | Ensembl Protists (Downloads); Adhikari et al. 2013; PLoS One 8: e75072 | par_scaffolds_v1 |
| *Phytophthora*  *infestans* T30-4 | genome (protein sequences) | NCBI GenBank; Haas et al. 2009; Nature 461: 393–398 | PRJNA49677 PRJNA17665 |
| *Phytophthora*  *parasitica* INRA-310 | genome (protein sequences) | NCBI GenBank | PRJNA259235PRJNA73155 |
| *Phytophthora*  *parasitica* INRA-310 | genome (cDNA sequences) | Ensembl Protists (Downloads) | PP_INRA-310_V2 |
| *Plasmopara halstedii* OS-Ph8-99-BlA4 | genome (protein sequences) | NCBI GenBank; Sharma et al. 2015; BMC Genomics 16:741 | PRJNA314514PRJEB6932 |
| *Plasmopara halstedii* OS-Ph8-99-BlA4 | genome (cDNA sequences) | Ensembl Protists (Downloads); Sharma et al. 2015; BMC Genomics 16:741 | Plasmopara_halstedii_genome |
| *Hylaoperonospora*  *arabidopsidis*Emoy2 | genome (cDNA & protein sequences) | Ensembl Protists (Downloads); Baxter et al. 2010; Science 330:1549-1551 | HyaAraEmoy2_2.0 |
| *Bremia lactucae* SF5 | Genome (protein sequences) | NCBI GenBank; Fletcher et al. 2019; Nat Commun 10:2645 | PRJNA387613 |
| *Phytophthora*  *lateralis* MPF4 | genome (protein sequences) | Ensembl Protists (Downloads); Quinn et al. 2013; FEMS Microbiol Lett 344:179-185 | MPF4_v1.0 |
| *Phytophthora*  *ramorum* Pr102 | genome (protein sequences) | Ensembl Protists (Downloads); Tyler et al. 2006; Science 313:1261-1266 | ASM14973v1 |
| *Phytophthora sojae*P6497 | genome (protein sequences) | NCBI GenBank; Tyler et al. 2006; Science 313:1261-1266 | PRJNA262907PRJNA17989 |
| *Phytophthora sojae*P6497 | genome (cDNA sequences) | Ensembl Protists (Downloads); Tyler et al. 2006; Science 313:1261-1266 | P_sojae_V3_0 |
| *Phytophthora kernoviae* 00238/432 | genome (protein sequences) | Ensembl Protists (Downloads); Sambles et al. 2015; Genom Data 6:193-194 | PhyKer238_432v1 |
| *Phytopythium vexans* (previously *Pythium vexans*) DAOM BR484 | genome (protein sequences) | Ensembl Protists (Downloads); Adhikari et al. 2013; PLoS One 8: e75072 | pve_scaffolds_v1 |
| *S. sapeloensis* | transcriptome *de novo* assembly | NCBI GenBank;  de Vries et al. 2019; BioRxiv, doi: doi.org/10.1101/656496 | PRJNA487262 |
| *Albugo laibachii*  Al Nc14 | genome (cDNA & protein sequences) | Ensembl Protists (Downloads); Kemen et al. 2011; PLoS Biol 9:e1001094 | ENA1 |
| *Albugo candida*  Ac Nc02 | genome (cDNA & protein sequences) | Ensembl Protists (Downloads) | ASM107853v1 |
