# Supplementary material for: A Global Survey of Carbohydrate Esterase Families 1 and 10 in Oomycetes
Source: Front Genet. 2020 Aug 7;11:756. doi: 10.3389/fgene.2020.00756 (PMC7427535; doi:10.3389/fgene.2020.00756)
Supplement: Supplementary file 2 [file Table_2.docx]

**Table S2. Metadata for oomycetes.**

| Species | lifestyle | Ref. | Host | Order |
| --- | --- | --- | --- | --- |
| A. candida | biotroph | Fawke et al. 2015 | Plant | Albugonales |
| A. laibachii | biotroph | Fawke et al. 2015 | Plant | Albugonales |
| H. arabdiopsidis | biotroph | Fawke et al. 2015 | Plant | Peronosporales |
| P. halstedii | biotroph | Fawke et al. 2015 | Plant | Peronosporales |
| B. lactucae | biotroph |  | Plant | Peronosporales |
| A. euteiches | necrotroph | Hughes and Grau 2007 | Plant | Saprolegniales |
| P. infestans | hemibiotroph | Fawke et al. 2015 | Plant | Peronosporales |
| P. parasitica | hemibiotroph | Fawke et al. 2015 | Plant | Peronosporales |
| P. sojae | hemibiotroph | Fawke et al. 2015 | Plant | Peronosporales |
| P. ramorum | hemibiotroph | Fawke et al. 2015 | Plant | Peronosporales |
| P. kernoviae | hemibiotroph | Judelson 2012 | Plant | Peronosporales |
| P. lateralis | hemibiotroph | Judelson 2012 | Plant | Peronosporales |
| P. vexans | necrotroph | Judelson 2012 | Plant | Peronosporales |
| P. iwayamai | necrotroph | Judelson 2012 | Plant | Peronosporales |
| P. aphanidermatum | necrotroph | Judelson 2012 | Plant | Peronosporales |
| P. arrhenomanes | necrotroph | Judelson 2012 | Plant | Peronosporales |
| P. irregulare | necrotroph | Judelson 2012 | Plant | Peronosporales |
| P. ultimum | necrotroph | Fawke et al. 2015 | Plant | Peronosporales |
| S. sapeloensis | saprotroph | Hulvey et al. 2010 | None | Peronosporales |
| S. diclina | necrotroph | Judelson 2012 | Animal | Saprolegniales |
| S. parasitica | necrotroph | Judelson 2012 | Animal | Saprolegniales |
| A. astaci | necrotroph | Judelson 2012 | Animal | Saprolegniales |
| A. invadans | necrotroph | Judelson 2012 | Animal | Saprolegniales |
| A. hypogyna | necrotroph | Judelson 2012 | Animal | Saprolegniales |
| T. clavata | saprotroph | Misner et al. 2015 | none | Saprolegniales |
| A. stellatus | unknown/opportunistic. pathogen | - | Plant | Saprolegniales |
|  |  |  |  |  |
