# Supplementary material for: A Global Survey of Carbohydrate Esterase Families 1 and 10 in Oomycetes
Source: Front Genet. 2020 Aug 7;11:756. doi: 10.3389/fgene.2020.00756 (PMC7427535; doi:10.3389/fgene.2020.00756)
Supplement: Supplementary file 3 [file Table_3.DOCX]

**Table S3. Transcriptomic datasets used in this study.**

| **Organism** | **Type of data** | **Dataset description** | **Reference** | **Accession/**  **Availability** |
| --- | --- | --- | --- | --- |
| *Albugo laibachii* Al Nc14 | Transcriptome reads | Inoculation of *Arabidopsis thaliana* leaves 8dpi | NCBI GenBank; Prince et al. 2017; BMC Biol 15:20 | PRJNA302221 |
| *Hyaloperonspora*  *arabidopsidis* Waco9 | Transcriptome reads | Inoculation of *Arabidopsis thaliana* leaves 5dpi | NCBI GenBank; Asai et al. 2014; PLoS Pathog 10: e1004443 | PRJNA232536 |
| *Phytophthora*  *infestans* 1306 | Transcriptome reads | Growing mycelium and inoculation of *Solanum tuberosum* tuber late phase | Ah-Fong et al. 2017; BMC Genomics 18:764 | PRJNA407960 |
| *Phytophthora parasitica* Pp016/INRA-310 | Transcriptome reads | Growing mycelium and inoculation of *Arabidopsis thaliana* roots 24hpi | NCBI GenBank | PRJNA382499  PRJNA168272 |
| *Plasmopara halstedii* OS-Ph8-99-BlA4 | Transcriptome reads | Infection of sunflowers | NCBI GenBank; Sharma et al. 2015; BMC Genomics 16:741 | PRJEB6932 |
| *Pythium ultimum* potato isolate from San Jacinto, CA | Transcriptome reads | Growing mycelium and inoculation of *Solanum tuberosum* tuber late phase | Ah-Fong et al. 2017; BMC Genomics 18:764 | PRJNA407960 |
| *S. sapeloensis* | Transcriptome reads | Growing mycelium and colonization of *Spartinia alterniflora* leaf litter 7dpi | NCBI GenBank;  de Vries et al. 2019; BioRxiv, doi: doi.org/10.1101/656496 | PRJNA487262 |
| *Aphanomyces euteiches*  ATCC201684 | Transcriptome reads | Growing mycelium and inoculation *Medicago truncatula* roots 9dpi | AphanoDB;  Gaulin et al. 2018 | PRJNA431223 |
| *Aphanomyces invadans* | Transcriptome reads | Growing mycelium | NCBI GenBank; | PRJNA188083 |
| *Aphanomyces astaci* | Transcriptome reads | Growing mycelium | NCBI GenBank; | PRJNA187375 |
| *Saprolegnia diclina* | Transcriptome reads | Growing mycelium | NCBI GenBank; | PRJNA182526 |
| *Saprolegnia*  *parasitica* CBS 223.65 | Transcriptome reads | Growing mycelium and inoculation of trout cell line 24hpi | NCBI GenBank;  Jiang et al. 2013; PLoS Genet 9: e1003272 | PRJNA164643 |
