# Supplementary material for: A Global Survey of Carbohydrate Esterase Families 1 and 10 in Oomycetes
Source: Front Genet. 2020 Aug 7;11:756. doi: 10.3389/fgene.2020.00756 (PMC7427535; doi:10.3389/fgene.2020.00756)
Supplement: Supplementary file 10 [file Image_1.PDF]

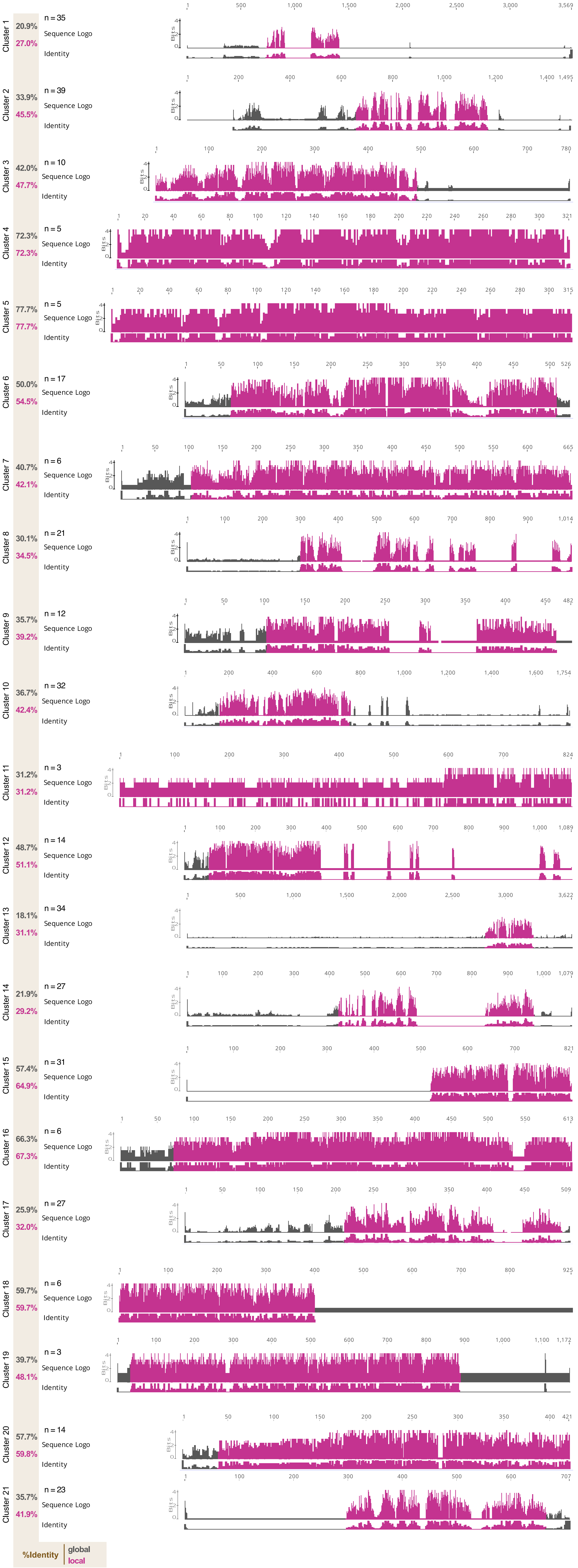

Figure S1. Alignment of the CE1 sequence clusters. All CE1 protein sequences of a clusters (1 to 21) were aligned using MAFFT with the G-INS-I settings, resulting in 21 alignments. The number of sequences in a cluster ("n =") are given on the left. The sequence fingerprint ("sequence logo") and a sliding window-based plot of the identity of these 21 alignments are depicted on the right. In the highlighted text on left, the global identity is given in grey font and the local identity is given in pink font; the pink colour of the alignment plots highlights the conserved region used for calculating the local identity. Note that sometimes the entire sequence was used for calculating the local identity due to its high conservation throughout the alignment.
